# Positioning justice: a legal cascade of sexual violence cases in Mombasa, Kenya

**DOI:** 10.3389/fgwh.2025.1605612

**Published:** 2025-07-10

**Authors:** Melanie Olum, Gerald Githinji, Abigael Sidi, Abel Mokua, Morris Kiio, Nawal Aliyan, Iqbal Khandwala, Celina Kithinji, Saida Wanjiku, Griffins Manguro, Marleen Temmerman

**Affiliations:** ^1^ Programs Department, International Centre for Reproductive Health—Kenya (ICRHK), Mombasa, Kenya; ^2^ Gender Based Violence and Recovery Centre (GBVRC), Coast General Teaching and Referral Hospital (CGTRH), Mombasa, Kenya; ^3^ Department of Health, Mombasa County (MOH, Mombasa County), Mombasa, Kenya; ^4^ Faculty of Medicine and Health Sciences, Ghent University, Ghent, Belgium; ^5^ Centre of Excellence in Women and Child Health, Aga Khan University (AKU), Mombasa, Kenya

**Keywords:** sexual violence, defilement, legal outcomes, paralegals, gender-based violence, gender-based violence recovery centre

## Abstract

**Introduction:**

Seeking legal redress for sexual violence (SV) is a daunting process for survivors. They must navigate their personal trauma societal stigma, and flawed systems, creating an arduous path to justice. This study was conducted to assess patterns of SV within Mombasa County, Kenya, and details the legal follow-up post-SV care.

**Methods:**

A retrospective cross-sectional study was conducted using data from SV survivors attending the Gender-Based Violence and Recovery Centre (GBVRC) at the Coast General Teaching and Referral Hospital (CGTRH) between 2017 and 2023. Data sources include post-rape care (PRC) forms, clinician notes, quarterly reports, police reports, counsellor reports, and paralegal follow-up records. A legal cascade for SV cases was created detailing SV survivors' referral patterns between police and GBVRC attendees who proceeded to court, and judgments rendered.

**Results:**

The total number of survivors at GBVRC between 2017 and 2023 was 3122; 2738 girls/women and 384 boys/men, with the majority of perpetrators known to the survivors, 2764/3122 (89%). The median age for survivors was 15 years, ranging from less than one year to 63 years with a male median age of 9 and a female median age of 15. Fewer males reported their cases, with 238 out of 384 males (62%) and 2,020 out of 2,738 females (74%). Ninety-three per cent (2906/3122) of cases referred from GBVRC arrived at the police station, though 62% (1864/3022) of these cases were successfully recorded and filed at the police station after follow-up. Additionally, 29% (535/1864) of cases reported did not proceed with investigation. Overall, of the 3022 cases that were referred to and reported, 1746 (57.7%) proceeded to court. Among these, judgments were pronounced in 372 cases (21%) and 85 out of these 372 cases (23%) were either withdrawn or acquitted.

**Discussion:**

Lack of evidence, poor witness testimonies, or failure of witnesses to present themselves in court were likely causes for withdrawal and acquittal of cases. Sexual violence remains a prevalent concern and should be prioritized as a national agenda. Systems should be strengthened to enhance access to justice while exploring alternatives for legal support, such as paralegal networks that can improve case follow-up.

## 1 Introduction

Sexual violence (SV) is a worldwide phenomenon and public health concern that bears physical, mental, emotional, and in some cases, economic effects on survivors. Sexual violence encompasses unwanted acts that range from verbal harassment to forced penetration, and an array of types of coercion, from social pressure and intimidation to physical force (1). It covers several activities ranging from rape to other physically less intrusive attempted or completed sexual contact. It typically embodies a lack of consent; the use of physical force, coercion, deception, or threat (1). SV results in a wide range of health problems in the immediate and long-term scope. These include physical injuries, Human Immunodeficiency Virus (HIV), sexually transmitted infections (STIs), unintended pregnancies, mental health conditions such as depression and anxiety, and sexual dysfunction in later life (2).

Globally, one in three women aged 15–49 years experienced physical or sexual violence mainly from a person well-known to them (3). Whereas global prevalence of sexual violence among men may be difficult to establish, reports have indicated that 1 in 10 men have experienced sexual violence in their childhood (4). The 2013 UN multi-country study further revealed that SV prevalence rates among adult men aged 18–49 years can range from 6% to 23% depending on the country (5) with higher rates of upto 24% observed in conflict zones (6). In Kenya, the 2019 Violence Against Children (VAC) survey reported that about 32% of girls and 16% of boys experience sexual violence before the age of 18 years (7). A 2018 study on school-related gender-based violence in Kenya further denoted that 68% of female students and 32% of male students experienced some form of school-related gender-based violence (8). A 2019 study on an Integrated Care Model for Survivors of Sexual Violence at the GBVRC at Coast Provincial General Hospital, Mombasa, Kenya also showcased that the majority of sexual violence survivors were under the age of 16 years and in every ten cases, eight were by perpetrators well known to the survivor (9).

Rooted in gender inequality, women disproportionately experience sexual violence. In addition, due to socioeconomic imbalances, women have fewer options and autonomy in their education, finances, and employment opportunities (10). Sexual violence among adolescents and children is also provoked by society's inability to recognize and protect their rights (11).

Access to justice by SV survivors is limited in low-resource settings (12). In different communities, a culture of tolerance and silence on sexual violence, in addition to power relations that undermine the position of women in society, discourage families from reporting sexual violence (13) where according to the VAC study, only one in ten of all survivors of sexual violence reported it to someone (7). Other factors which discourage survivors from reporting include stigma, survivor blaming, and fear of retaliation where the perpetrators are well known to the survivors (9). Limited proximity to reporting sites and non-prosecution of sex offenders also contributes to low reporting will among survivors (13).

Kenya has several laws and frameworks guiding preventative and responsive GBV measures, such as the National Constitution 2010, The Children Act No. 8 of 2001, and more recently the Children's Act 2022 among others. Specifically, the National Policy for Prevention and Response to Gender-Based Violence is cognizant of the need for concrete and efficient systems to operationalize laws and plans for effective GBV prevention and response. The 2014 policy was designed to hasten attempts towards coordinated approaches in GBV programming, enforcement of laws, and advanced access to quality, comprehensive care (14). While these policies and frameworks offer a national backdrop for GBV prevention and response, county-specific strategies that are evidence-driven are equally important.

Successful SV prevention strategies require comprehensive documentation on the trends in SV (clinical, psychosocial, and legal) to inform prevention practice (15). Clinical management of SV has been documented by numerous studies, detailing the expectations and loopholes within health facilities, including gender-based violence and recovery centers (16). However, there is limited documentation on the legal follow-up of SV cases and challenges that deter access to justice at each stage of the legal process. Focus has often been on healthcare, but there is a need for a comprehensive approach to include a legal rights perspective. The study is a follow-up to our previous 2018 study on an integrated care model for survivors of SV (9), intending to detail the component of legal follow-up further. This study, therefore, sought to explore and report on the legal structures and cascade for SV cases in Mombasa County, Kenya.

## 2 Materials and methods

### 2.1 Study design

This is a retrospective cross-sectional study using data from the GBVRC at the Coast General Teaching and Referral Hospital (CGTRH). We retrospectively extracted data from all SV survivors’ records at the CGTRH-GBVRC between January 2017 and December 2023. This is a follow-up analysis to our previous study (9) that catered to data covering the years previous to 2017. We further reviewed literature from the paralegal reports, quarterly project reports, and clinician notes to obtain data records on legal follow-up.

### 2.2 Intervention

The GBVRC provides an integrated post-SV service–delivery model within a government referral facility catering to clinical, psychosocial, and legal services (9). Briefly, the Centre was established in 2007 as a collaboration between the International Centre for Reproductive Health Kenya (ICRHK), CGTRH, and Mombasa County. ICRHK is a non-government research organization that undertakes research and interventions on reproductive health in Kenya. It operates with varied stakeholders, including the National and County governments.

The GBVR Centre was established as a model Centre, as well a training and research site, as part of a public-private partnership between the three institutions. The Centre has provided care to 9,846 survivors since its inception in 2007. Care in this case refers to clinical management, psychosocial support, and legal support. Survivors are both males and females of different ages, from as young as 1 year old to more than 50 years old.

The GBVRC adapts a systematic case management process for survivors. Once a survivor reports to the Centre, they receive clinical services from a trained clinician. Details of the incident are entered into the post-rape care (PRC) form, which is a standard Ministry of Health (MOH 365) examination documentation form. In this study, data were extracted from PRC forms from 2017 to 2023. The PRC form is filled in for every survivor presenting at the GBVRC and is used for both medical and legal purposes. It documents the socio-demographic characteristics of the survivor, the nature of the sexual violence act, where it occurred and when, who the perpetrator was, the physical examination findings on presentation, investigations done, results of the investigations, treatment given, and referral to any other services if any. The clinician also maintains a record of additional case notes for detailed reporting. The PRC form also collects information on police reporting, such as whether the survivor reported the assault before coming to the GBVRC.

Further to emergency post-SV clinical care, the Centre provides psychosocial support through counseling services and legal support through 5 paralegals attached to the Centre. It facilitates linkages with the police, the judiciary, local leaders, and the community at large all under specific processes and procedures. Survivors are offered psychosocial support by a trained trauma counsellor who provides counseling sessions. Ideally, all survivors should undergo five counselling sessions carried out via a national counselling guide. This is according to global and national prescribed standard operating procedures. A record of counselling notes is created with each visit.

Five paralegals are engaged as volunteers, although they receive a modest stipend equal to the stipend for community health volunteers in Kenya (approximately USD 40 or 5,000 Kenya Shillings per month). This is catered for by the various funders supporting operations at the centre. After clinical care, they accompany survivors to the police station and help them navigate services. They may be present as the survivors fill in the Police Reporting form 3 (P3) form. Subsequently, the paralegals visit the police stations each month, collect data on the process of each of the reported cases, and follow up where necessary. They then prepare a summary report of cases referred to court post investigation and obtain a detailed account of those that are pending at the stations. They also remind the survivors of court appointment dates and, if necessary, accompany survivors to their court date hearings where they can stand as witnesses. One paralegal is attached to one of six police stations within the catchment area of the GBVRC.

### 2.3 Data collection

Data from survivors who were either self-referrals or referred from other departments, including the police, or health facilities to the GBVRC, was extracted from the program database, namely post-rape care (PRC) forms, clinician notes, GBVRC quarterly reports, counsellor reports, and paralegal follow-up records. In our study, the key variables captured from the PRC included: survivor demographics (Table 1) and police reporting patterns. Table 2 presents the police reporting patterns of the survivors, while Table 3 highlights the perpetrator characteristics. We collected all perpetrator data available on the PRC namely the gender, age, familiarity and profession. We only analysed data on age and gender because data on profession was not clear, as it relied on recall from survivors, often affected by trauma. We undertook an in-depth review of the paralegal reports and quarterly project reports to extract data on the cases referred from police to the GBVRC, cases referred to police after service at the GBVRC, the status of cases at the police, and the status of cases at the courts. This is available in Table 4.

**Table 1:** Demographic data of sexual violence survivors at the GBVRC from 2017 to 2023 (*N* = 3,122).

Year of assault		2017	2018	2019	2020	2021	2022	2023	Overall
*n*		303	525	544	462	460	509	319	3,122
Sex	Male *n* (%)	25 (8)	70 (13)	66 (12)	61 (13)	62 (13)	55 (11)	45 (14)	384 (12)
	Female *n* (%)	278 (92)	455 (87)	478 (88)	401 (87)	398 (87)	454 (89)	274 (86)	2738 (88)
Summary statistics	Median	14	14	14	14	15	15	15	15
	Median age female (Min-Max)	14 (0-52)	15 (0–50)	15 (0–63)	15 (0–48)	16 (0–57)	9 (1–50)	15 (1–63)	15 (0–63)
	Median age male (Min-Max)	8 (4–35)	10 (2–40)	8 (0–43)	9 (1–40)	11 (0–36)	915 (1–40)	8 (1–38)	9 (0–43)
Age group of survivors *n* (%)									
0–5 years	Male	6 (24)	13 (19)	20 (30)	11 (18)	12 (19)	4 (7)	9 (20)	75 (32)
	Female	26 (9)	34 (7)	51 (11)	49 (12)	40 (10)	50 (11)	33 (12)	283 (9)
6–10 years	Male	11 (44)	25 (36)	25 (38)	31 (51)	18 (29)	30 (55)	20 (44)	160 (5)
	Female	58 (21)	71 (16)	63 (13)	48 (12)	43 (11)	55 (12)	32 (12)	370 (12)
11–14 years	Male	3 (12)	21 (30)	13 (20)	8 (13)	17 (27)	10 (18)	4 (9)	76 (2)
	Female	68 (24)	109 (24)	105 (22)	75 (19)	76 (19)	85 (19)	63 (23)	581 (19)
15–17 years	Male	3 (12)	6 (9)	6 (9)	4 (7)	7 (11)	6 (11)	3 (7)	35 (1)
	Female	68 (24)	143 (31)	155 (32)	159 (40)	157 (39)	193 (43)	109 (40)	984 (32)
18–25 years	Male	1 (4)	3 (4)	1 (2)	5 (8)	4 (6)	3 (5)	7 (16)	24 (1)
	Female	32 (12)	57 (13)	66 (14)	49 (12)	53 (13)	46 (10)	24 (9)	327 (10)
26 and above	Male	1 (4)	2 (3)	1 (2)	2 (3)	4 (6)	2 (4)	2 (4)	14 (0)
	Female	26 (9)	41 (9)	38 (8)	21 (5)	29 (7)	25 (6)	13 (5)	193 (6)

Source: PRC Form GBVRC.

**Table 2:** Reporting patterns of sexual violence survivors at the GBVRC from 2017 to 2023 (*N* 3,122).

Referral patterns		2017	2018	2019	2020	2021	2022	2023	Overall
Perpetrator known to the survivor *n* (%)	Female	247 (89)	376 (83)	400 (84)	373 (93)	362 (91)	407 (90)	245 (77)	2,410 (88)
	Male	25 (100)	59 (84)	62 (94)	57 (93)	56 (90)	50 (91)	45 (100)	354 (92)
	Overall	272 (90)	435 (83)	462 (85)	430 (93)	418 (91)	457 (90)	290 (91)	2,764 (89)
Reported to police before GBVRC	Male	15 (60)	40 (57)	43 (65)	36 (59)	41 (66)	35 (64)	28 (62)	238 (62)
	Female	207 (74)	344 (76)	356 (74)	287 (72)	302 (76)	330 (73)	194 (71)	2,020 (74)
	All	222 (73)	384 (73)	399 (73)	323 (70)	343 (75)	365 (72)	222 (70)	2,258 (72)
Timing for reporting the assault to police	within a day	7 (3)	108 (28)	166 (42)	139 (43)	113 (33)	100 (27)	44 (20)	579 (26)
	more than one day but within a week	1 (0)	39 (10)	50 (13)	41 (13)	45 (13)	58 (16)	33 (15)	267 (12)
	more than a week but within a month	0 (0)	14 (4)	10 (3)	14 (4)	17 (5)	24 (7)	10 (5)	89 (4)
	over a month	1 (0)	5 (1)	11 (3)	17 (5)	47 (14)	258 (71)	197 (89)	536 (24)
	Date of reporting and/or date of assault not indicated-	294 (97)	359 (68)	307 (56)	251 (54)	238 (52)	69 (19)	35 (16)	1,553 (69)
Police follow-up post-referral (police confirming, collecting and signing PRC form)	No	15 (5)	51 (10)	34 (6)	55 (12)	39 (8)	18 (4)	4 (1)	216 (7)
	Yes	288 (95)	474 (90)	510 (94)	407 (88)	421 (92)	491 (96)	315 (99)	2,906 (93)

Source: PRC Form GBVRC.

**Table 3:** Perpetrators’ characteristics as per cases received at the GBVRC from 2017 to 2023.

Perpetrators characteristics *n* (%)	Period	2017	2018	2019	2020	2021	2022	2023	Total
Sex of perpetrator	Male	269 (99)	425 (98)	448 (97)	386 (90)	398 (95)	50 (96)	28 (97)	2,004 (96)
	Female	3 (1)	10 (2)	14 (3)	44 (10)	20 (5)	2 (4)	1 (3)	94 (4)
Age categories	≦10	7 (2)	10 (2)	13 (2)	17 (4)	9 (2)	15 (3)	12 (4)	83 (3)
	11–15	16 (5)	42 (8)	37 (7)	26 (6)	26 (6)	27 (5)	17 (5)	191 (6)
	16–20	55 (18)	121 (23)	120 (22)	111 (24)	131 (28)	172 (34)	118 (37)	828 (27)
	21–25	44 (15)	60 (11)	77 (14)	56 (12)	59 (13)	63 (12)	39 (12)	398 (13)
	26–30	66 (22)	79 (15)	115 (21)	81 (18)	88 (19)	104 (20)	43 (13)	576 (18)
	>30	80 (26)	128 (24)	124 (23)	85 (18)	95 (21)	102 (20)	67 (21)	681 (22)
	Unknown	24 (8)	43 (8)	38 (7)	6 (1)	0 (0)	23 (5)	9 (3)	143 (5)
	Missing	11 (4)	42 (8)	20 (4)	80 (17)	52 (11)	3 (1)	14 (4)	222 (7)

Source: PRC Form GBVRC.

**Table 4:** Follow-up of cases at the police station and in courts from 2017 to 2023 (*N* = 3,122).

Year		2017	2018	2019	2020	2021	2022	2023	Total
*n*		303	525	544	462	460	509	319	3,122
Total new cases in contact with the police *n* (%)		295 (97)	506 (96)	525 (97)	439 (95)	446 (97)	495 (97)	316 (99)	3,022 (97)
Status of cases at the police station (including previous years)									
	Cases at the police station(s)	295	424	481	148	110	87	319	1,864
Status of cases in courts	Cases under investigation	^a^	404 (95)	246 (51)	147 (99)	75 (68)	^b^226	231 (72)	1,329 (71)
	Cases in court (*n*)	62	204	183	375	318	261	343	1,746 (94)
	Sentencing (5–30 years)	9 (15)	5 (2)	19 (10)	10 (3)	73 (23)	25 (10)	62 (18)	203 (11)
	Life imprisonment	4 (6)	2 (1)	9 (5)	6 (2)	0 (0)	19 (7)	33 (10)	73 (4)
	Referred to the high courts	0 (0)	0 (0)	0 (0)	2 (1)	0 (0)	0 (0)	0 (0)	2 (0.1)
	Acquittals	1 (2)	1 (0)	25 (14)	2 (1)	3 (1)	14 (5)	8 (2)	54 (3)
	withdrawn	0 (0)	2 (1)	1 (1)	4 (1)	0 (0)	21 (8)	3 (1)	31 (2)
	Probation	0 (0)	1 (0)	0 (0)	0 (0)	0 (0)	7 (3)	1 (0)	9 (0.5)
Total judgments made		14 (23)	11 (5)	54 (30)	24 (6)	76 (24)	86 (33)	107 (31)	372 (21)

Source: paralegal follow-up reports.

^a^ Reporting on cases under investigation at the GBVRC commenced in 2018.

^b^ In 2022 the GBV Court In Shanzu Kilifi County was launched which caused a push in processing of cases that may have been pending over the years. This court caters to cases in Mombasa and the wider coastal region.

### 2.4 Data management

Data from the PRC forms was entered into an online database created by ICRHK. The online database with PRC records was managed by ICRHK and was stored on an encrypted server. Access to this server was only by authorized individuals. Hard copies of the PRC forms were stored in a lockable cabinet. The counsellor reports were recorded onto a separate online database housed on encrypted ICRHK drives. The clinician and paralegal notes/reports were kept in a separate lockable unit at the GBVRC. The clinician, paralegal, and counsellor notes were extracted and compiled into monthly and quarterly reports submitted to the hospital and ICRHK Program Managers where they were used to optimize program implementation. Data verification and cleaning were continuously done to ensure the records were up-to-date and accurate.

### 2.5 Data analysis

**Figure 1:**
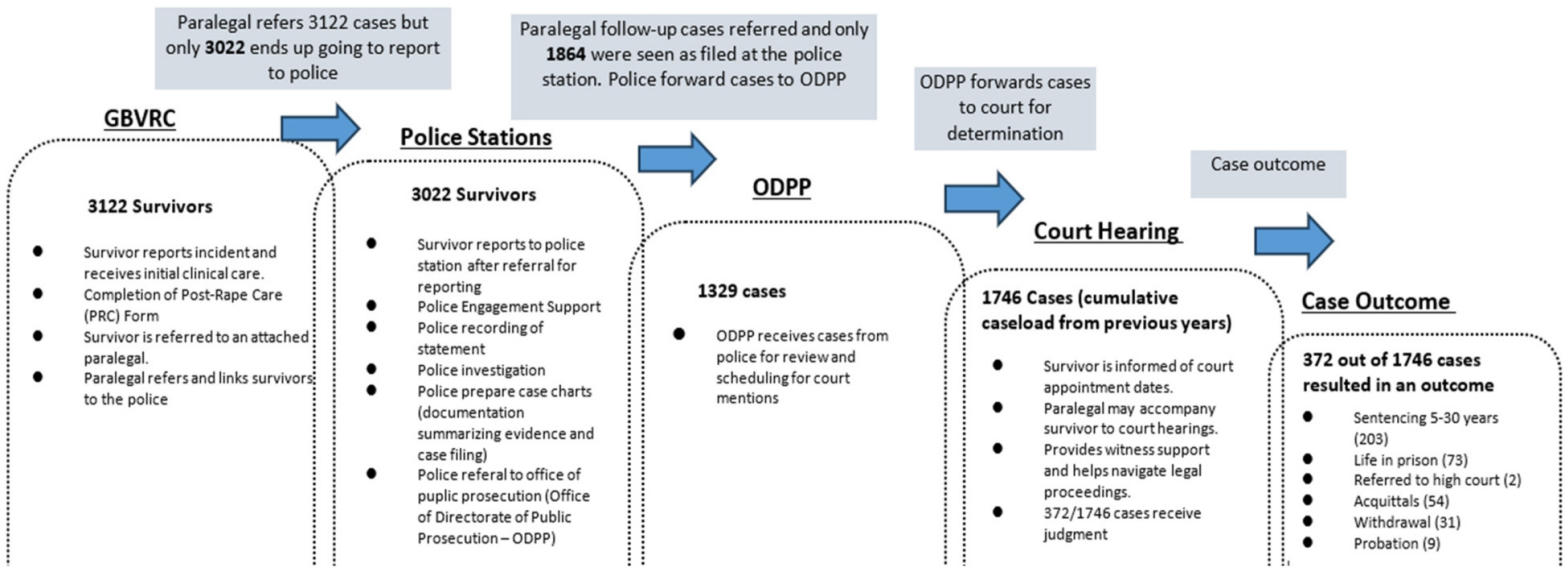
Legal cascade of case reporting, follow-up and processing.

Descriptive statistics were generated from all persons/survivors included in the analysis. Continuous variables were summarized in the form of means with standard deviation, while categorical variables were summed up as proportions. We created a legal cascade for sexual violence cases (Figure 1), including the number of survivors who received care at the Centre, those who were referred to the police, the number who eventually ended up reporting to the police station, cases that went to court, and the number of judgments that have already been rendered. We have abridged the types of decisions in the results section.

### 2.6 Ethical considerations

The study involved secondary analysis of previously collected data through data abstraction. Data was de-identified before analysis to safeguard the identities of the survivors. No direct contact was made with participants to minimize risk. All data was stored in encrypted files, accessible by select study staff who followed protocol, as per data confidentiality training and agreements. We obtained ethics approval from the AMREF Ethics Review Committee (ESRC P1532/2023) and the Coast General Teaching and Referral Hospital Ethics Review Committee (ERC-CGH/Msc./VOL.I), which reviewed the study protocol and approved the publication of findings. National research approvals were secured through the National Commission for Science, Technology & Innovation (NACOSTI) (NACOSTI/P/24/33596).

## 3 Results

### 3.1 Socio-demographic information of survivors

Table 1 summarizes demographic and assault characteristics of all survivors (*n* = 3122) who received care at the Centre and who were included in the analysis, disaggregated by gender with annual trends spanning from January 2017 to December 2023. On average, 15 survivors received care at the Centre each week. The median age was 15 years, with majority aged 15–17 years (33%). Among female survivors, the median age was 15 years, while it was 9 years for the male survivors, with 62% aged below 10 years. Female survivors between 0 and 10 years accounted for 25% of all female cases, and 72% were under 17 years, indicating a high burden on SV among young adolescents (Table 1).

In about 89% of the cases, the perpetrator was well-known to the survivor, with a higher percentage of familiarity among male survivors (92%). Community level reporting was high, where in 72% of cases, the survivor had already reported to the police station before coming to the GBVRC. Moreover, out of all cases with known reporting times, over half (846/1,471) reported within a week of the incident. There appeared to be a strong linkage between the GBVRC and the police, with 72% of the cases that ended up at the GBVRC being referred by the police. Moreover, follow-up of cases showed that 93% (2,906/3,122) of cases referred from the GBVRC did arrive at the police station (Table 2).

### 3.2 Perpetrators' characteristics

Table 3 presents the gender and age categories of the perpetrator (s). Most perpetrators were male (96%), with over half (58%) within the age category of 16–30 years.

### 3.3 Reporting and judgement patterns

Retention of cases along the cascade, particularly between the police and the courts, appeared to be the most challenging (Figure 1). Out of the cases referred to and reported at the police station, 62% (1,864/3,022) of these cases were successfully recorded and filed at the police station after follow-up, which is a 38% drop in cases, which were lost between referral and filing. Further drops in cases were seen, where 29% (535/1,864) of cases reported did not proceed with investigation (Table 4).

Delays in judgment were also observed. Out of all cases presented to the courts, only 21% (372/1,746) resulted in a judgment over the 7 years. There were notable fluctuations in judgment rates across the years, with 23% in 2017, 5% in 2018, 30% in 2019, 6% in 2020, 24% in 2021, 33% in 2022, and 31% in 2023. Various sentences were pronounced, and 55% (203/372) resulted in 5 to 30-year sentencing. Life imprisonment was at 20% (73/372) and 23% (85/372) were acquitted or withdrawn, likely due to lack of evidence, poor witness testimonies, or failure of witnesses to present themselves in court (Table 4).

## 4 Discussion

This study explored the assault characteristics and reporting patterns of survivors attended to at the GBVRC CGTRH from the year 2017 to 2023, along with the legal outcome of the cases. The majority of the survivors were children aged 15–17 years, and most perpetrators were known to the survivors, with over half of perpetrators aged 16–30 years. Case reporting at the police station was high, though cases proceeding to court were almost half the number reported. A fifth of the reported cases proceeded to court and had judgments pronounced, with over half of the cases resulting in a sentencing of 5–30 years. Trends in judgments made appeared to be fluctuating across the years, with the highest dip being between 2019 and 2020. The lack of evidence, poor witness testimonies, or failure of witnesses to present themselves in court were causes for the withdrawal and acquittal of some of the cases, which is expected given the prevailing realities of out-of-court settlements, intimidation and mishandling of evidence that reportedly affect the country's judicial system (17).

This study's finding that the majority of survivors were 14 years old corroborates other studies that indicate a higher prevalence of SV among adolescents aged 12–16 years (9, 18). Consistent with other research that place high victimisation of sexual violence in Africa among adolescent girls and young women (19, 20), this study further presents that most survivors were females under the age of 17 years confirming that the burden of sexual violence in the country like other low and middle income countries (LMICs) is on young adolescents and women. Kenya recently rolled out a triple threat campaign to reduce the prevalence of three interconnected threats affecting adolescent girls that is GBV, HIV infections and teenage pregnancies (21). These study findings suggest a need for greater emphasis on tackling adolescent health concerns among younger adolescent girls under 17 years who are at a higher risk of sexual assault, particularly in countries that are not highly ranked in gender equality—Kenya is currently ranked 135 out of 139 (22). This would require targeted efforts to address inequitable gender, social, political, and economic norms in order to reduce the subordination of women and vulnerable groups in society (23).

Study results further acknowledge the prevalence of sexual violence among males, where the age group of 0–10 years accounted for 61% of male survivor cases. Interestingly, from the age categories of 11 years onwards, there are fewer male survivors, which is consistent with another 2020 Médecins Sans Frontières (MSF) study that posited a higher proportion of child male survivors compared to other age groups, including adolescents aged 12–17 years (24). This may suggest that younger males are more vulnerable than their older male counterparts, but this could also suggest reluctance in reporting among older males due to stigma or victimisation where they fear being ridiculed, emasculated, and questioned for their sexuality (25, 26). Studies show that male survivors may adopt several post-trauma coping mechanisms such as self-blame, drug abuse, withdrawal, and at times reckless/harmful sexual behaviour (27), which could encourage a cycle of abuse where survivors could become offenders (28). Ignoring trends in sexual abuse among males, who also grapple with gendered narratives that affect how they cope with the trauma, could therefore further ignite an upsurge of sexual violence and inhibit progress towards addressing the vice (29). Alternatively, Thomas and Kopel (30) suggest that appreciating the impact of victimisation on reporting among both males and females can increase and encourage more survivors to report cases of sexual violence. Recognizing and validating the experiences of male survivors may encourage them to come forward, seek help and participate in sexual violence prevention and support efforts.

Transfer and prosecution of cases along the legal cascade appear to be the main challenge in sexual violence case management. Though the use of a paralegal system at the GBVRC-CGTRH may account for 97% of case reporting, during the follow-up process, a notable drop of 29% (535/1,864) of cases under investigation at the police station was recorded. This suggests a challenge in handling cases at the police station, which could result from survivor intimidation leading to non-followup, poor documentation and mismanagement of evidence; all of which compromise the integrity of cases filed (31).

Moreover, out of the 94% of cases that proceeded to court, only 21% resulted in a judgment, eliciting further concerns about case prosecution within the court system. The study showed that 23% of cases filed at the court were acquitted and withdrawn, potentially due to fear of retaliation, threats, payoffs, and stigma often experienced by survivors, especially where the perpetrator is well known (32). Social norms that blame survivors for the assault or those that prioritize family honor over the survivor's well-being also discourage follow-up of sexual violence cases among survivors (33). Such occurrences pose a significant challenge to achieving better legal outcomes for survivors and deter ongoing efforts towards curbing violence in the community.

The 21% of judgments made across the years further suggest a drag in sexual violence case management, which was also reflected in the national COVAW 2022 study, where out of the 3,791 sexual offences cases filed within a selection of 7 courts from 2017 to 2021 in Kenya, only 37% were officially closed (32). Adjournments, unsynchronised court diaries, non-appearances of medical doctors and investigating officers, missing files, scanty investigations, limited support for vulnerable witnesses, unavailability of witnesses, a lack of specialized courts, and the transfer of officers were cited as the key causes of delayed judgments as per the report which may explain the lag as seen in this study (32).

Limited coordination between family and criminal courts, the reluctance of the police to investigate cases and the limited availability of resources within the court system have also been cited as some additional inhibitors to justice for sexual violence survivors (12). National legislation in many developing countries is often inferior to traditional law and community-level justice systems that possess greater cultural authority. Subsequently, informal punishments such as pay-offs to the survivor's family or marrying off the survivor to the perpetrator are common, ultimately compromising legislative enactments on sexual violence crimes (34). At the law enforcement level, once cases have been reported to the police station, there is mismanagement of files, misconduct among officers, negative community interference and intimidation. Collectively, this may result in the dropping of charges by the survivors and their families (30), potentially explaining the 38% drop in cases referred to the police and those filed at the police station across the years.

Several potential reasons for the drop in cases under investigation have been highlighted above, along with reasons for the delay in judgment. Although only 21% of judgments were made, this is still higher than in some African countries, where, in South Africa, for example, only 340 (8.6%) cases were finalised with a guilty verdict (35, 36). This suggests the potential of a well-coordinated paralegal approach in case management that supports follow-up and tracking from reporting to determination of judgment.

Despite the advantageous ranking of 21% judgment compared to other countries, there were fluctuating trends in judgment across the years, with notable plunges in judgments between 2017 and 2018 (18%), 2018 and 2019 (25%), and 2019 to 2020 (24%). The change in regime in Kenya between 2017 and 2018 may have affected the drop of 18% in judgments within that period, further positing a lack of independence within the judicial system, where country politics can potentially interrupt the flow of legal services by changing the legal systems, leadership and administration (37). Judicial adaptations may have also influenced the change in trends over the years. According to the “Rule of Law in the time of COVID-19: Kenya, 2020” report, the courts were closed at the onset of the pandemic, which may explain the drop in 24% of judgments made between 2019 and 2020. However, a 19% increase in judgments observed in 2022 may be attributed to the implementation of courtroom digitization, introduced to sustain operations Covid-19 crisis (38). This digital shift has been infused into Kenya's judicial system to minimize delays and enhance efficiency in court proceedings. Judicial adaptations are therefore not only necessary in improving case management in routine cases but can have an extensive impact during unforeseen disruptions such as a pandemic.

### 4.1 Implications for policy and practice

Kenya has laws and policies designed to protect persons against sexual exploitation and or violence such as The National Constitution 2010, The Children Act No. 8 of 2001, and more recently the Children's Act 2022, Sexual Offences Act No. 3 of 2006, and The Victim Protection Act, No. 17 of 2014 among others. Their implementation is, however, difficult owing to financial constraints, legal pluralism, cultural beliefs, legal illiteracy, poor competence among some service providers, and inadequate monitoring frameworks (23). Greater investment is therefore required to reform the legal system, making it more survivor-centred to encourage increased reporting, ensure sustainable case management, and support the timely prosecution of cases. Complete independence of the judicial and legal system is integral to ensure transparent case management and justice for survivors. Regime change in many LMIC settings has been seen to destabilise systems and structures, including at the judicial level, by sparking shifts in legal frameworks and judicial appointments (37). There is a need, therefore, for more sustainable judicial systems that are independent and adequately integrated to uphold the rule of law and ensure timely access to justice.

### 4.2 Recommendations

Sexual violence remains a prevalent concern and should be prioritized as a political agenda for collaborative and inclusive efforts that extend beyond policy formulation to effective implementation. Kenya appears committed to addressing GBV, as is evidenced by the launch of twelve specialized GBV courts across the country. With the onset of the specialized courts, it is expected that the conviction of GBV cases is likely to increase in the years to come. A comparative look at Uganda, where similar courts were introduced in 2018, shows an increase in conviction rates from 20% to 70% (39). Although such legal reforms are necessary to enhance access to justice among SV survivors, they represent an initial step towards more systemic change. This paper recommends specific measures and further research to improve legal outcomes as highlighted:

• establishing additional GBV courts across all counties in Kenya and possibly hiring more magistrates to reduce backlog in SV cases.
• Maintaining a clear schedule of hearings between the prosecution office and the courts to support timely review of cases and prevent unnecessary adjournments.
• Capacity building of law enforcement for enhanced professionalism and transparent investigations and upholding the integrity of evidence submitted.
• Enforcing structured consultation between law enforcement and the office of public prosecution to reduce instances of improper case documentation.
• Develop an integrated system that links cases reported, filed and investigated at the police station and those filed at the prosecutor's office to improve tracking of cases along the legal cascade
• Establish stronger paralegal networks across counties for better case follow-up.
• Explore the role and experiences of males as perpetrators, survivors and agents of change in sexual violence. This is important because boys who are violated in their childhood can become perpetrators later in life. Additionally, males are integral to addressing gender narratives and imbalances that perpetuate violence.

### 4.3 Study limitations

This study is subject to certain limitations. For instance, clinical service data is ideally not a research representative of a real-world setting. Proof of concept from routine clinical services can however be important for research. The data were obtained retrospectively and are likely to have some omissions at different data points. Some factors may be presented that were not originally measured. There is also no individual-level data, thus making it difficult to link this information to the judicial systems within the County. Additionally, self-reported data of this nature is prone to introspective ability bias. Survivors may grapple with accurate awareness of their behavior and responses due to trauma, which may affect the accuracy of self-reported data. Lastly, the data is only limited to the GBVRC at CGTRH; hence the study findings are not representative of Kenya. Despite the limitations, this study possesses some strengths that can inform future research and interventions. Data was captured over a long period and can therefore be used to deduce comparisons.

## 5 Conclusion

In this research, younger females under 17 years bear the highest burden of sexual violence, though younger males under 11 years accounted for most of the male survivors. Though community-level reporting was high and there were strong linkages between the GBVRC and the police, there were notable challenges in the retention of cases along the legal cascade, with a drop between cases reported and those filed for investigation. Further delays in judgment were observed across the years, which may be due to systemic shortcomings suggesting shortcomings within law enforcement and the judicial system. This study presented a legal cascade of SV cases in Mombasa, while positioning several challenges in case management within the region. We sought to highlight the legal component which, though often overlooked, is integral to comprehensive care for SV survivors. We anticipate that these findings will advise sexual violence programming within the County and inform legislative and community-level interventions on legal follow-up and security.

## Data Availability

The data analyzed in this study is subject to the following licenses/restrictions: https://figshare.com/articles/dataset/Legal_Paper_Data_v2_xlsx/28722983&lt;/b&gt. Requests to access these datasets should be directed to akothmelanie@gmail.com.
